# Advances in Aptamer Drug Delivery Systems for Treatment of Glioblastoma

**DOI:** 10.1002/advs.77811

**Published:** 2026-09-27

**Authors:** Alexandra R. Paul, Alexandra Moreno Mitchell, Dean O'Neil, Yutong Luo, Khuloud T. Al‐Jamal

**Affiliations:** ^1^ Department of Pharmacology and Pharmacy LKS Faculty of Medicine The University of Hong Kong Hong Kong SAR China; ^2^ Institute of Pharmaceutical Science Faculty of Life Sciences & Medicine King's College London London UK

**Keywords:** aptamer, blood brain barrier, drug delivery, glioblastoma, targeting

## Abstract

Therapeutic strategies for glioblastoma have progressed considerably over recent decades. Despite the development of new biomedical technologies and advances in our understanding of physiology and disease progression, effective drug delivery to brain cancer remains a significant challenge. The challenge is delivering therapeutics to the correct location without losing therapeutics to non‐desirable cells as well as overcoming the low permeability posed by the blood‐brain barrier. This could be tackled by conjugating a specific targeting agent to the therapeutic or drug delivery system to guide, such as an aptamer. Aptamers are short, single‐stranded oligonucleotides with high selectivity and specificity for their targets, making them primed for a vast array of diagnostic and therapeutic applications. Aptamers are powerful molecular tools with the potential to overcome many of the challenges inherent in treating brain‐based diseases. Here is discussed the use of aptamers, specifically as drug delivery vehicles, for the treatment of glioblastoma. There are a multitude of drug delivery aptamers in development, each with their own optimized formulation and drug delivery strategy. This review systematically and comprehensively details every drug delivery system that includes aptamers as a glioblastoma therapy that has been published to date.

## Graphical abstract

Aptamers have remarkable recognition capacity which is suitable for assisting drug delivery for GB. This review demonstrates that aptamers have great potential as targeted therapeutic carriers, conjugated to various therapeutic carriers, using a range of conjugation strategies and chemistries. These aptamer conjugates are able to cross the BBB and target the tumor successfully with many producing successful therapeutic outcomes in GB models.

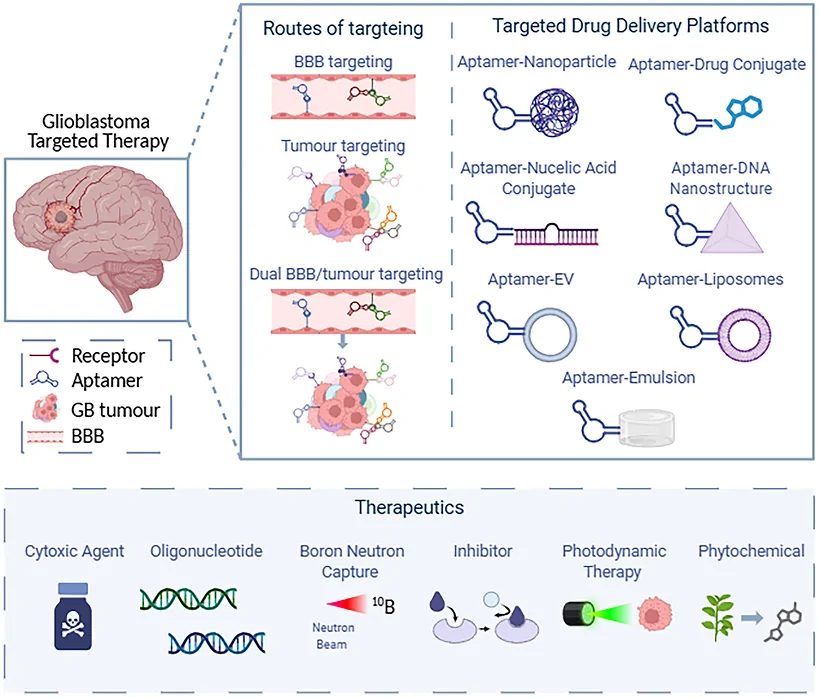

**Abbreviations**
BNCT: boron neutron capture therapy
GB: Glioblastoma
MMAE: monomethyl auristatin E
NP: nanoparticle
PDGFR‐β: Platelet derived growth factor receptor β
SELEX: Systematic Evolution of Ligands by EXponential Enrichment
TAM: tumor‐associated macrophages
TfR: transferrin receptor

## 1 Introduction

### 1.1 Glioblastoma

Glioblastoma (GB) is a grade IV primary brain cancer that arises from glial cells [1]. GB is the most common malignant brain tumor in adults, accounting for 45.2% of primary malignant central nervous system (CNS) tumors. GB is the most fatal CNS malignancy, with a median survival rate of 15 months following diagnosis [2]. GB is more common in men than women and increases with age, with the most commonly affected age range being between 75 to 85 years old [3]. Risk factors associated with GB include ionizing radiation and a genetic susceptibility caused by mutations in specific gene expressions for example, TP53, DH1, EGFR [4]. Diagnosis of GB occurs through a combination of magnetic resonance imaging (MRI) and histopathological analysis of surgically excised tumor biopsies [2]. Since 2005, the standard care for GB has been invasive surgical resection, followed by radiotherapy and temozolomide chemotherapy [5]. Other than temozolomide there are four drugs and one device FDA‐approved for the treatment of high‐grade gliomas such as lomustine, intravenous carmustine, carmustine wafer implants, bevacizumab and tumor treatment fields (TTFields). These treatments are FDA approved; however, they have not been universally approved and are not a part of the standard of care yet [6]. The highly infiltrative nature of GB tumors is the leading cause of recurrence, as complete surgical resection is often unlikely. The heterogeneous complexity of GB tumors is attributed to the poor success of many modern treatments [7]. Given the poor survival with currently approved treatments for GB, new therapeutic strategies are urgently needed. In recent years, advancements in immunotherapy and nanotechnology have demonstrated potential to expand GB treatments, however, the blood‐brain barrier (BBB) remains a challenge for drug delivery. Nanoparticle (NP) delivery platforms are becoming increasingly popular for encapsulating therapeutics for better delivery, but a challenge that is faced is delivering the NPs to the correct locations and losing therapeutics to non‐desirable cells. Using a specific targeting agent could be a way to tackle off targeting, by guiding the NP to the desired cells. The use of aptamers as a/in a drug delivery vehicle for GB targeting has been newly investigated to help overcome the shortcomings of traditional treatments such as targeting the tumor directly and crossing the BBB [8].

### 1.2 Aptamers

Aptamers are single‐stranded oligonucleotides that mimic monoclonal antibodies (mABs) by folding into complex 3D shapes that bind non‐covalently with high affinity and specificity to diverse targets [9]. Oligonucleotides are short nucleic acid molecules (∼15–60 nucleotides long) generated by solid‐phase phosphoramidite chemistry [10]. So far, aptamers have been selected against viruses [11], proteins [12], polysaccharides [13], bacteria [14], toxins [15], peptides [16], small molecules [17], amino acids [18] and whole cells [19]. The recognition capacity of aptamers can be harnessed for therapeutic agents, drug‐delivery targeted ligands and diagnostic applications. The name ‘aptamer’ was first introduced in 1990 [20] originating from the Latin word ‘aptus’ meaning ‘to fit’ and the Greek word ‘meros’ meaning ‘part’ [21].

Systematic Evolution of Ligands by EXponential enrichment (SELEX) is a commonly used method to identify oligonucleotides with high affinity for a specific target molecule. Frequently utilized in aptamer selection, SELEX relies on a large and random assortment of oligonucleotides (10^14–^ 10^16^) to determine DNA/RNA sequences that best bind to a chosen target. The standard in vitro SELEX method consists of immobilization of the target molecule on a solid support [22] or use of a cell line, followed by incubation with the oligonucleotide library. The non‐binding sequences are then washed away, and bound sequences are separated from their target, amplified by polymerase chain reaction (PCR), and retained for the next selection cycle; negative selection cycles are normally added to help avoid non‐specific binding. Usually, 6–15 rounds of SELEX will be completed before the aptamer candidates are identified by sequencing [23], subsequently followed by further analysis such as computational docking, stability, toxicity and binding affinity studies [24]. In addition, there may also be post modifications made to aptamers to increase affinity, stability or allow for conjugation to another molecule.

The protocol for the generation of aptamers is simple; however, it is a time‐consuming and labor‐intensive process. Aptamer selection by SELEX does not always have the best affinity and specificity due to a sub‐optimal SELEX procedure. New methods have been designed to reduce these limitations such as automated SELEX (microfluidic‐based SELEX) [25, 26], capillary electrophoresis SELEX (CE‐SELEX), Selection of Optimized Ligands by Fluorescence‐Activated Bead Sorting (SOLFABS) [27, 28], cell‐based SELEX [29], in vivo SELEX [29] and high‐throughput sequencing SELEX (HT‐SELEX) [30] (Table 1). CE‐SELEX includes a modified stage of selection. Non‐equilibrium capillary electrophoresis of equilibrium mixtures is used for aptamer fractioning, a procedure that takes 1–2 days and allows for more stringent selection of aptamers that bind to a chosen target [31, 32]. SOLFABS is a method that combines high‐precision automated flow cytometry‐based selection with an enzyme‐free sequence readout; as PCR amplification is not used, all selections can be done on the same day. This method also allows for chemical modifications of the aptamer library, generating aptamers with greater stability and higher affinity [27]. Microfluidic‐based SELEX enables the processing of small fluid volumes and the rapid generation of aptamers with high affinity. Innovations such as micro‐magnetic separation devices and microarray‐integrated microfluidic chips have increased the efficiency and specificity of this approach [30]. Cell‐based SELEX uses whole live cells as targets, enabling aptamers to interact with molecular targets in their native conformation and successfully select for specificity against biomarkers under physiological conditions [33].

**TABLE 1:** Strengths and weaknesses of different types of SELEX: Protein SELEX, Cell‐SELEX, CE‐SELEX, SOLFABs, Automated‐SELEX, HT‐SELEX, in vivo SELEX. Each SELEX method is described in terms of their advantages or disadvantages of being used to select aptamers for GB.

SELEX	Key point	Strengths	Weaknesses	Application in GB aptamer selection
Protein SELEX	Aptamer selection for recombinant protein	Target known Quicker and easier than other types of SELEX	Protein is not in its native state Potentially will not translate to in vivo, as the binding was not under physiological conditions.	Good if wanting to select for a target specific to GB or BBB. The protein will not be in its native state, this may not translate over to the true BBB or tumor environment.
Cell‐SELEX	Aptamer selection for whole live cell	Aptamers can recognize whole parts of the cell's surface. Binds to cells in native state and under physiological conditions.	Lengthy procedure, 8–20 rounds will be completed. Needs further target identification. Target bias, as the aptamer will bind to the most abundant protein.	Good if wanting to select for a GB cell specifically. The cell will not be in its native state, the aptamer may not be able to cross the BBB to reach the tumor.
CE‐SELEX	Uses the strong electric fields of capillary tubes to separate binding from non‐binding aptamers	Faster method, cuts down the rounds to 2–4. Less material is used. Can be used on non‐immobilized targets.	Equipment is expensive. Target limited, it works poorly for whole cells. Specialist training for equipment.	Will produce aptamer candidates fasters but no guaranty they will work in the GB environment.
SOLFABs	Aptamer selecting using Fluorescence‐Activated Bead Sorting method	No PCR needed, causing no loss of potential sequences and no PCR sequence errors. All aptamer selections can be achieved in 1 day. Can be used for cells or protein. Improved binding affinity with modified aptamers	Specialist equipment and training required. Complicated sequence determination method.	Using a modified aptamer library will increase stability and selectivity. Still limited on protein or cell selection.
Automated SELEX	Microfluidic‐based SELEX	Faster method, cuts down the rounds to 3–5. Low reagent use. High stringency	Expensive set up cost Operating the microfluidic chip introduced technical complexity. Smaller library size	Will produce aptamer candidates fasters Could set up the microfluidics to mimic the GB environment
HT‐SELEX	SELEX that used a deeper sequencing method	Tracks millions of sequences in parallel, unlike traditional Sanger sequencing. Tracks millions of sequences in parallel, unlike traditional Sanger sequencing, which only reads a few dozen.	Next generation sequencing is expensive Data overload—It is hard to tell if a highly frequent sequence is a great binder or just a result of PCR duplication or library synthesis errors.	Still limited on protein, cell or animal selection. Will provide more accurate sequences to trial.
In vivo SELEX	SELEX inside living organisms, used to find aptamers that bind to a specially organ.	Highly relevant, as aptamers can withstand real physiological conditions. Biological stability. Discover real targets.	Expensive set up costs Complex process requiring expert skills. Biological noise, unwanted sequences will be introduced from the host.	A GB tumor bearing animal will give an accurate environment for aptamer selection. Very complicated process producing large biological noise.

#### 1.2.1 Aptamers as Therapeutics

Aptamers have the potential to treat diseases that currently have no cure. There are several ways in which aptamers can do this: act as a drug delivery agent, an agonist or an antagonist. Aptamers can act as an agonist by binding and subsequently activating cell‐surface receptors. They can act as antagonists by inhibiting protein‐protein interactions or receptor‐ligand interactions [10]. The first aptamer approved for therapeutic use by the USA Food and Drug Administration (FDA), was in 2004 and called pegaptanib. It is an RNA aptamer that consists of 28 nucleotides [34] and has been routinely used to treat macular degeneration [35]. Pegaptanib (commercial name Macugen) is provided as an injectable solution and has been shown to improve the vision acuity of adults with the wet form of age‐related macular degeneration. Pegaptanib has a 2’fluorophyridine modification that does not interfere with reverse transcriptase mediated amplification [36].

Aptamers can be used for therapeutic purposes in a similar way to antibodies. Antibody therapy is a form of immunotherapy that uses mABs to bind to certain cellular and extracellular proteins, ultimately stimulating the immune system to attack targeted cells [37]. Despite their proven track record, aptamers have several advantages over protein therapeutics in terms of synthetic accessibility, modifications and size [9]. Targeting proteins in extracellular or cell‐surface targets removes the need for the therapeutics to cross the cell membrane [10], effectively circumventing the protective lipid bilayer. Theoretically, aptamers can be used as therapeutics in any disease for which extracellular blockade of protein‐protein interaction is needed [38].

There are other oligonucleotide‐based therapies approved for therapeutic use or undergoing clinical trials. Currently, there are several antisense oligonucleotide‐based drugs that have entered phase III clinical trials such as Alicaforsen, ALN‐TTR02, and QPI‐1002 [39]. Alicaforsen (identified using SELEX technology) works by inhibiting the production of ICAM‐1, a cell surface receptor involved in the process of inflammation. Alicaforsen binds to ICAM‐1 mRNA, effectively reducing the expression of ICAM‐1. Clinical trials of Alicaforsen have shown reduced levels of inflammation, a reduction in symptoms for the patient, and an improvement in the health of the tissue shown by endoscopy [40].

#### 1.2.2 Aptamer as Diagnostics

The key to effective treatment is an accurate and early diagnosis. Effective early diagnosis is only possible if the method is sensitive and involves small detecting agents, making aptamers ideal candidates. Detectable signals can be generated by ligand‐mediated conformational changes in an aptamer, providing a highly sensitive platform amenable to diagnostic applications. The ease of conjugation and labelling aptamers also allows them to be combined with other advanced technologies such as flow cytometry [41], microfluidics [42], cell separation [43], endogenous nucleic acid analysis [44] and nanoparticle‐based sensing [45]. All these qualities make aptamers a great potential tool for diagnostics [46].

Aptamers have been used for detection of parasital [47], bacterial [48], and viral‐mediated [49] diseases and cancer. Some existing diagnostic methods require expensive equipment and skilled operators, leading to high costs [46]. To reduce costs, researchers have identified DNA aptamers selected against cancer cells, which can be utilized to image cancer tissue for more cost‐effective diagnosis [50]. Biomarkers that are specifically expressed or overexpressed on cancer cells can be used in selection of diagnostic DNA aptamers; cells from liver, colon, pancreatic, prostate, and breast cancer have all been used to evaluate this novel approach. Example biomarkers are VEGF [51], prostate‐specific membrane antigen (PSMA) [52] and epidermal growth factor receptor variant III (EGFRvIII) [53].

Aptamers can be modified as a diagnostic tool for parasite disease. Minopoli et al. developed an ultrasensitive plasmon‐enhanced fluorescence biosensor for detecting Plasmodium falciparum lactate dehydrogenase (PfLDH), a malaria biomarker, in whole blood [54]. The biosensor uses a combination of mABs and aptamers for specific detection. It achieved a femtomolar limit of detection without requiring sample pretreatment, offering a significantly more sensitive alternative to traditional malaria diagnostic tests. Within the context of bacterial disease, a DNA aptamer was developed to bind and detect non‐pathogenic Sterne strain Bacillus anthracis spores [55], utilizing an aptamer‐magnetic beads‐electrochemiluminescence sandwich assay scheme for detection. High‐affinity spore surface‐bound aptamers were detected by their 5'‐biotinylated tails using labelled avidin [56]. Chen et al. developed a DNA aptamer‐based method for detecting the SARS‐CoV‐2 nucleocapsid protein, which could significantly enhance the accuracy and speed of COVID‐19 diagnosis. This approach offers high affinity binding to the nucleocapsid protein, providing a potential alternative to traditional PCR methods and possibly paving the way for more rapid testing protocols [57].

## 2 Aptamers for Targeting Glioblastoma

As well as serving as direct therapeutics or as a diagnostic, aptamers have also been developed as drug delivery vehicles or used as targeting ligands in drug delivery vehicles [58]. Aptamers functioning as targeted drug delivery carriers or targeting ligands may increase the efficacy of a drug and reduce the destructive side effects of other non‐targeted approaches, such as systemic chemotherapy. As using aptamers as therapeutics themselves for GB has not been highly successful, potentially using aptamers for their high selectivity and binding affinity skills will be a better alternative.

From the research carried out for the use of aptamers targeting GB, there are two approaches being used, targeting receptors in the tumor microenvironment for a specific delivery of therapeutics or targeting the BBB to allow the delivery system to pass through to the tumor. The BBB, even though considered ‘leaky’ at the tumor core, is still intact at the infiltrating edge, meaning tumor cells are still protected [59]. Some of the studies are using these two approaches dually, to allow for BBB passing and tumor specific targeting simultaneously (Figure 1). The targets chosen by researchers are CD133, nucleolin, U‐87 tumor cells, patient derived glioblastoma stem cells, transferrin, human platelet derived growth factor receptor β. As these aptamers used are generally for targeting and not therapeutic agents the targets are protein that are over expressed on the tumor or the BBB, are general GB cells lines or GB cells with the target overly expressed or are ex vivo GB patient samples.

**FIGURE 1:**
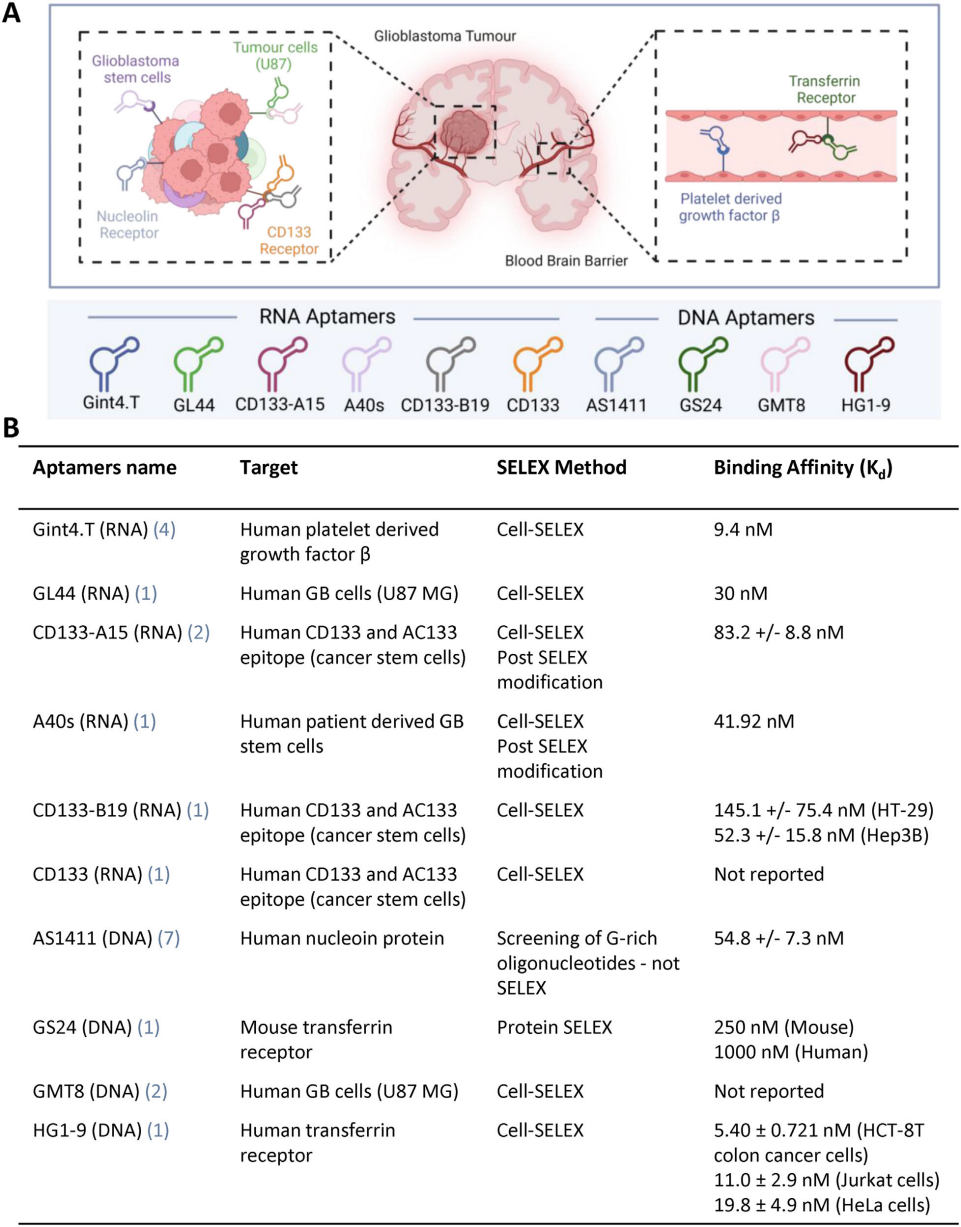
Aptamers for targeting glioblastoma. (A) Schematic of aptamers used as targeting ligands for glioblastoma targeting and crossing the BBB. (B) Table of aptamers used in glioblastoma research with their corresponding targets SELEX method and K_d_ values. Number in blue represents the number of times this aptamer is used in GB drug delivery systems.

### 2.1 Tumor Aptamer Targets

#### 2.1.1 CD133 Receptor

CD133 (known as Prominin‐1) is a membrane‐bound glycoprotein on the surface of cancer stem cells (CSCs) and has been associated with tumor cell proliferation, tumorigenesis, therapy resistance, poor prognosis, and tumor recurrence in GB. Recent studies have shown the connection between CD133 and key signaling pathways such as PI3K/AKT, WNT/b‐catenin, and Notch, which manages the cell cycle checkpoints and tumor progression [60]. CD133 receptor is regarded as an attractive and relevant target for GB therapy, especially as it acts as a marker for GB stem cells being expressed on 10–70% of cells [61, 62]. This is a popular target for aptamer targeting in GB with 3 different RNA aptamers being selected for the protein including CD133‐A15 aptamer, CD133 aptamer and CD133‐B19 aptamer over 4 different studies. All three aptamers have the same origin; they were selected against HEK293T cells that express CD133 [63]. CD133‐A15 aptamer is 15 nucleotides long with a K_d_ value of 83.2 ± 8.8 nM, this aptamer appears in studies by Smiley et al. [64] and Sun et al. [65]. Smiley et al. conjugated the aptamer CD133‐A15 to a polymer‐micellar NP composed of poly(styrene‐b‐ethylene oxide) (PS‐b‐PEO) and poly(lactic*‐co‐*glycolic) acid (PLGA) co‐loaded with temozolomide and RG7388 therapies. The NP are tested in human GB CSCs with positive marker CD133, no animal studies were carried out in this study. Sun et al. uses a dual modified cationic liposome loaded with survivin siRNA and PTX. Liposomes were functionalized with a BBB lipoprotein receptor (angiopep‐2) along with the CD133‐A15 targeting aptamer. This delivery system was tested in U‐251 MG‐CD133+ tumor‐bearing mice. CD133 aptamer is 14 nucleotides long, originating from CD133‐A15 aptamer (minus one base). This aptamer appears in a study by Liang et al. [66] in a dual delivery system utilizing exosomes as the carrier and 2 targeting ligands: angio‐pep2 peptide to target the BBB (targeting low‐density lipoprotein receptor‐related protein 1 (LRP1)) and CD133 aptamer to target the tumor, delivering temozolomide and O6‐benzylguanine to U‐87 MG tumor‐bearing mice. CD133‐B19 aptamer targets both CD133 protein and AC133 epitope and is 19 nucleotides long. This aptamer appears in a study by Behrooz et al. [67] CD133‐B19 aptamer is conjugated to polyamidoamine (PAMAM) G4C12 dendrimer nanoparticles, formulated for the co‐delivery of paclitaxel and temozolomide to U‐87 MG stem cells, no animal therapy studies were carried out in this research. CD133 marker shows potential from these 4 studies as a good targeting strategy for GB therapy.

These studies have favored dual targeting and dual delivery using extra targeting tools alongside the targeting aptamers (angio‐pep2 peptide) to target the BBB and then the tumor, and delivering two types of drug, an anti‐tumor drug alongside another such as an inhibitor or silencing agent. All systems were a type of NP as the delivery vehicle. The main models used in these studies are the CD133 receptor, U‐87 MG (human GB cell line), U‐87 MG tumor bearing mouse model, U‐251 MG‐CD133+ (human GB cell line) tumor bearing mouse model and human GB cancer stem cells. These are the current best models that can be used for targeting CD133 protein.

#### 2.1.2 Nucleolin Receptor

Nucleolin is present on the cell surface of proliferating and tumor cells, shuttling from the nucleus to the plasma membrane, it is essential for cell proliferation [68]. Nucleolin has been shown to be overexpressed in GB patient brain samples compared to normal brain samples (up to 100% expression) and this is why it is a good target for a targeting ligand for brain delivery [69]. AS1411 is a guanine‐rich 26‐nucleotide long aptamer is a DNA aptamer with a K_d_ value of 54.8 ± 7.3 nM, this is a very popular aptamer for human nucleolin [70]. AS1411 was not developed by SELEX but discovered by chance [71]. It appears in 7 papers being used in drug delivery systems for GB. AS1411 has been used in a diverse range of drug delivery systems and testing models. Wang et al. [72] created a hyaluronic microemulsion system for dual drug delivery of Shikonin (SKN) & Docetaxel (DTX) using AS1411 as the targeting ligand. This system was tested in U‐87 MG/CSC spheroids followed by U‐87 MG tumor‐bearing mice. The same research team (Wang et al. [73]) created a different drug delivery system of a superparamagnetic Fe_3_O_4_ NP microemulsion loaded with the same drugs SKN and DTX. This time, functionalizing the system dually with AS1411 and T7 peptide (which targets transferrin receptor for BBB crossing targeting). They used a different animal model of G422 tumor‐bearing mice, which is a mouse cell line model, instead of the human cell line from their previous study. Luo et al. [74] used a AS1411 functionalized poly(L‐ γ ‐glutamyl‐glutamine) nanoconjugate with paclitaxel. This drug delivery system was tested on U‐87 MG spheroids and U‐87 MG tumor‐bearing mice. Fu et al. [75] designed a tetrahedral framework nucleic acid (tFNA) loaded with temozolomide. This system was dually conjugated with AS1411 aptamer and GS24 aptamer for transferrin receptor. They studied this system in 4 different cell lines A172, U‐87 MG, T98G and LN‐18. Healthy animal biodistribution studies were done; however, no animal therapy studies were completed in this study. Zhu et al. [76] developed mesoporous ruthenium nanoparticles loaded with anti‐tumor drug Ru(bpy)_2_(tip)]^2+^ (RBT) and conjugated with Transferrin and AS1411 aptamer. They used this platform in U‐87 MG cells, U‐87 MG sphere model and U‐87 MG tumor‐bearing mice. Sathiyaseelan et al. [77] designed 5‐Fluorouracil loaded Au nanoparticles conjugated with chitosan, loaded with doxorubicin and further functionalized with AS1411 aptamer. This delivery system was tested in LN229 cell line, no animal therapy studies were carried out in this study. Seo et al. [78] developed an AS1411 aptamer conjugated dsDNA streptavidin hybrid nanospheres with doxorubicin. They tested this drug delivery system in U‐87 MG cells, U‐251 MG cells, U‐87 MG spheroids and U‐87 tumor‐bearing mouse models.

AS1411 is a popular aptamer with great potential as being used as a targeting ligand for GB therapy. The drug delivery systems used for targeting nucleolin with AS1411 are very diverse, from different types of NPs to nucleic acid frameworks and microemulsions, using sometime dual targeting systems with dual therapy delivery. These systems for nucleolin as the target are used in various models such as LN229, U‐251 MG, A172, U‐87 MG, T98G, G244 and LN‐18 cell line, ex vivo HUVECs cells, transwell model hCMECs/U‐87 MG cells, U‐87 MG and U‐87 MG/CSCs spheroids, U‐87 MG and G422 tumor‐bearing mice and healthy BALB/c nude mice. This shows great versatility of AS1411 aptamer and its successful use across multiple cell lines and animal models.

#### 2.1.3 Tumor Cells (U‐87 MG)

U‐87 MG cell line is a widely used human malignant GB cell line which was derived from a 44‐year‐old female patient. U‐87 MG is a good GB model because it can produce orthotopic brain models which are highly reproducible and have good growth. The tumors are vascularized, making them a great drug testing model [79]. Being a human cell line also helps with therapy translation from bench top to the clinic. GL44 is an RNA aptamer selected against U‐87 MG cells, 45 nucleotides long producing a K_d_ of 30 nM. GMT8 is a 75‐nucleotide long DNA aptamer, which selectively binds to U‐87 MG cells. Vorobyeva et al. [80] developed a *closo*‐dodecaborate for ^10^B isotope delivery for boron neutron capture therapy (BNCT) conjugated with GL44 aptamer. U‐87 MG was the model used in this study; no animal therapy studies were conducted in this research. Gao et al. [81] developed a poly (ethylene glycol)‐poly(ε‐caprolactone) (PEG‐PCL) nanoparticle loaded with Docetaxel and conjugated with GMT8. The models used were U‐87 MG cells, U‐87 MG 3D spheroids, U‐87 MG tumor‐bearing mouse model. Shi et al. [82] designed a tetrahedral framework nucleic acid (tFNA) loaded with paclitaxel and conjugated with aptamers GMT8 and Gint4.T (BBB aptamer). This study used models U‐87 MG cells and a transwell model including bEnd.3/U‐87 MG cells.

U‐87 MG is a popular GB model with other studies that do not include a specific U‐87 MG aptamers, showing making a specific aptamer for this cell line has great potential. The models used in this research all have the origin of U‐87 MG but in different forms such as U‐87 MG cell line, U‐87 MG 3D spheroids, transwell with U‐87 MG and bEnd.3 cells and U‐87 MG tumor‐bearing mice. Three diverse types of delivery systems were used for targeting U‐87 MG, aptamer‐drug conjugate, polymer NP and nucleic acid framework. GL44 and GMT8 aptamers bind to the surface of U‐87 MG cells without knowing which receptor they bind to, it would be interesting to identify the surface protein they bind to, to understand the treatment pathway at a deeper level.

#### 2.1.4 Patient Derived Glioblastoma Stem Cells

Affinito et al. [83] developed a chimera to deliver miRNA (miRNA‐34c and anti‐miRNA‐10b) using A40s aptamer. A40s aptamer targets patient‐derived GB stem cells (GSC), it is an RNA aptamer 30 nucleotides long with a K_d_ value of 41.92 nM. These A40s‐miRNA chimeras were tested back in the patient‐derived GSCs. It would be interesting to see this delivery platform tested in an animal model such as U‐87 MG. GSCs are a good GB target as they can cause tumor relapse and chemoresistance, which is a main role in GB recurrence and aggressiveness. However, the GSCs would potentially differ from patient to patient, testing this platform on multiple patient samples would give more conclusive data if this system was patient specific or not. Identifying the cell surface protein that the aptamer is specifically binding to could help with this, as if this protein is over expressed in all patients, then it could be used universally. The identification of the cell surface marker could also produce a new therapeutic to be explored in other ways.

### 2.2 Blood‐Brain Barrier Aptamer Targets

#### 2.2.1 Transferrin Receptor

The transferrin receptor (TfR) is a transmembrane glycoprotein, which is primarily TfR1 also known as CD71. It works by facilitating cellular iron uptake, which it does by binding iron loaded transferrin and internalizing it via endocytosis. TfR is a highly promising, extensively researched target for GB therapy. It is abundantly expressed on the brain capillary endothelial cells in the BBB and is known to be significantly upregulated in brain tumors. Because TfR1 is preferentially expressed on brain endothelial cells compared to peripheral tissues, it is heavily exploited as a biological gateway to shuttle large therapeutic molecules across the BBB for neurological diseases [84], producing targeted drug delivery via receptor‐mediated transcytosis [85]. TfR is significantly overexpressed to meet the aggressive metabolic and iron demands of the tumor cells that are rapidly dividing. This causes tumor progression by clathrin‐mediated endocytosis of iron‐laden transferrin [86]. GS24 and HG1‐9 are DNA aptamers that both selectively target the TfR, GS24 is specific targeting to mouse protein (however has some weaker binding to human cells) and HG1‐9 is human specific and will not bind to mouse cells [87]. GS24 is used in combination with AS1411 (seen in section 2.1.2, Fu et al. [75]). GS24 aptamer is 64 nucleotides long and has the binding affinity for mouse TfR at 250 nM and for human at 1000 nM. While being selected for mice, the models used for testing are all human. HG1‐9 is 35 nucleotides long and has binding affinity of 5.40 ± 0.721, 11.0 ± 2.9, and 19.8 ± 4.9 nM for HCT‐8T cells, Jurkat cells and HeLa cells, respectively. HG1‐9 is used in a study by Zhao et al. [88] where they created and aptamer drug conjugate by linking HG1‐9 and highly potent anti‐tubulin drug monomethyl auristatin E (MMAE). They explore this platform in the BBB transwell model of bEnd.3 cells together with U‐87 MG cells as well as U‐87 MG subcutaneous and orthotopic mouse models.

This promising data shows that TfR is a favorable target to be studied for GB therapy. As TfR is a BBB target the ideal models to use are a transwell model and in vivo tumor‐bearing mice models. These are to validate the systems passing through a BBB and arriving in the tumor. HG1‐9 model is tested in the BBB transwell model of bEnd.3/U‐87 MG cells as well as U‐87 MG subcutaneous and orthotopic mouse models, the AS1411/GS24 models should be tested in the same models, as testing just on a cell line does not give the real picture of the double targeting of BBB and tumor cells.

#### 2.2.2 Human Platelet Derived Growth Factor Receptor β

Platelet derived growth factor receptor β (PDGFR‐ β) is a receptor tyrosine kinase that binds to PDGF ligands, predominantly expressed on brain capillary pericytes [89]. It is used to regulate cell proliferation, migration and angiogenesis. It is a critical target for solid tumors as it is highly overexpressed, making it a good target for GB [90]. Gint4.T is a 38 nucleotide RNA aptamer that binds to human PDGFR‐β with a K_d_ of 9.6 nM, but has shown to also bind to the mouse protein. Gint4.T is a therapeutic and targeting drug delivery aptamer simultaneously. It works by inhibiting cell migration and acting as a delivery vehicle across the BBB [91]. Wang et al. [92] developed a DNA tetrahedron loaded with doxorubicin and conjugated with Gint4.T. Models used in this study are U‐87 MG cells, no animal therapy studies are conducted in this study. Monaco et al. [93] created polymeric nanoparticles (PNPs) with poly(lactic*‐co‐*glycolic)‐ block‐polyethylene glycol (PLGA‐b‐PEG) copolymer loaded with P13K‐mTOR inhibitor conjugated with Gint4.T. The system was tested in U‐87 MG cells and U‐87 MG tumor‐bearing mouse models. Fei et al. [91] created a Gint4.T‐siHDGF chimera‐capped mesoporous silica nanoparticle encapsulating temozolomide (TMSN@siHDGF‐Gint4). This system will deliver both temozolomide and siHDGF simultaneously. Models used in this study are U‐87 MG cells, U‐87 MG/bEnd.3 transwell system, U‐87 MG spheroids and U‐87 MG tumor‐bearing mice. Gint4.T was also used alongside GMT8 aptamer by Shi et al. [82] (section 2.1.3).

Using PDGFR‐ β as a receptor and Gint4.T as an aptamer for GB therapy had some good potential as seen by this data. Gint4.T in combination with a tumor cell specific aptamer would also make a good targeting pair. Gint4.T has mostly been used in nucleic acid frameworks and different types of NPs and the models used were U‐87 MG cell line, U‐87 MG/bEnd.3 transwell systems and U‐87 MG tumor‐bearing mice. These models are good for this type of tumor targeting.

## 3 Aptamers for Delivery in Glioblastoma

There are two main ways aptamers can be used in drug delivery systems. They can either be used as a targeting ligand, or they can be the drug delivery vehicle itself while also being a targeting ligand. The drug delivery systems explored for GB delivery are aptamer‐drug conjugates, aptamer‐nucleic acids, aptamer‐NPs, aptamer‐EVs, aptamer‐liposomes and aptamer emulsion (Figure 2).

**FIGURE 2:**
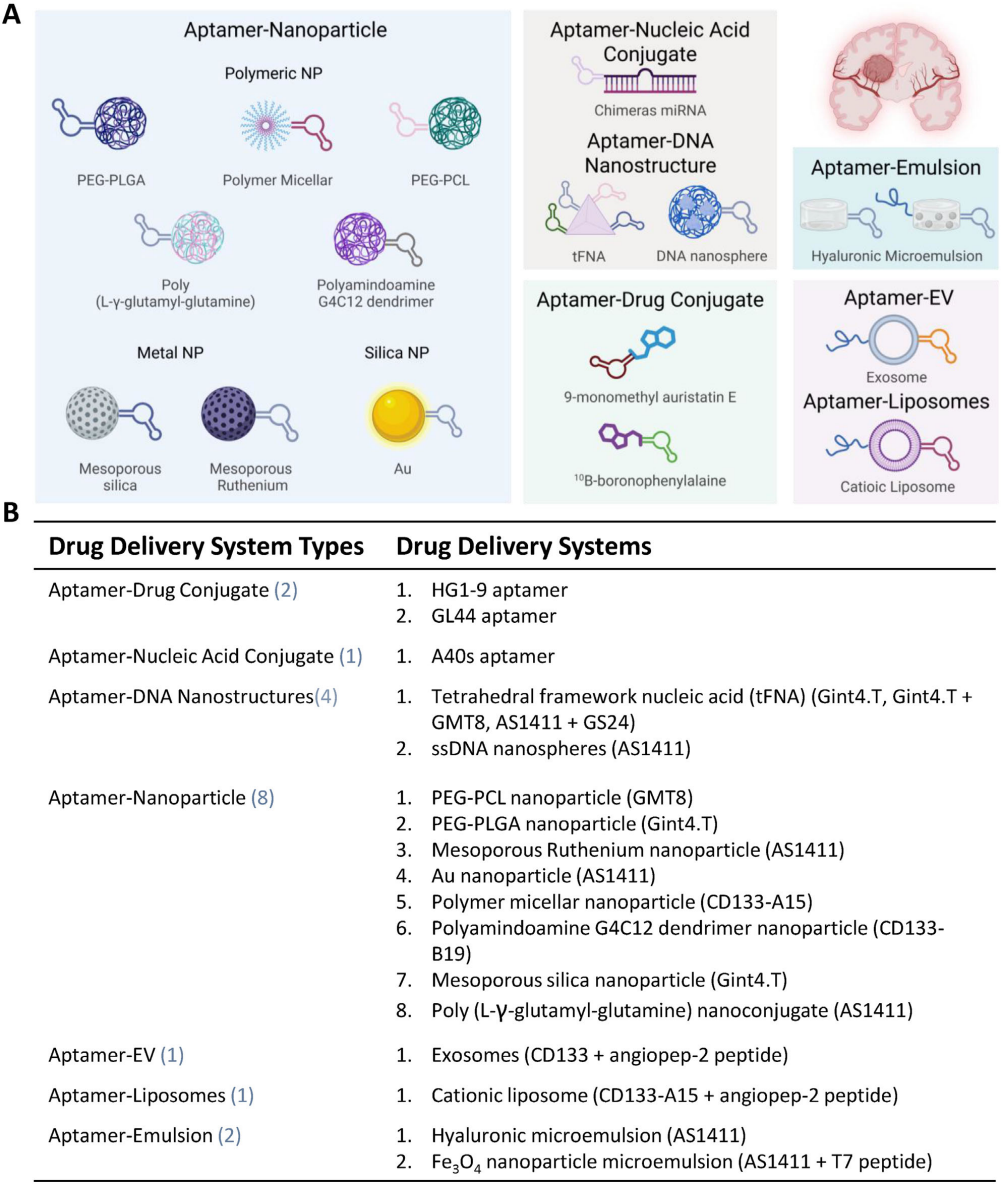
(A) Schematic of drug‐ or delivery system‐ aptamer conjugates used for glioblastoma therapy. (B) Table of type of drug delivery systems involving aptamers and their specific delivery system make up. Number in blue represents the number of times this drug delivery system is used in GB.

### 3.1 Aptamers as Delivery Systems

#### 3.1.1 Aptamer‐Drug Conjugate

Aptamer‐drug conjugates (ApDCs) represent a strategic approach in targeted therapy where aptamers are directly linked to therapeutic agents. Attaching drug molecules to one or more sides of the aptamer have been regarded as an effective solution to overcome the side effects caused by off‐target binding of drugs [46]. In the ApDCs, the aptamer functions as a precise targeting device that identifies and binds to specific targets. High‐affinity binding ensures that the conjugate delivers its therapeutic payload directly to the cells of choice whilst facilitating efficient internalization of the drug [94]. The ApDCs typically consist of three main components: the targeting molecule (aptamer), linker, and the therapeutic agent (drug) [95]. The incorporation of therapeutics onto aptamers is achieved through non‐covalent and covalent methods [96]. Non‐covalent methods utilize the distinctive characteristics of aptamers to attach drugs through interactions without forming stable bonds, such as electrostatic interactions, hydrophobic interactions, simple hybridization and intercalation [97]. Non‐covalent approaches enables temporary binding that can be modified or reversed under certain biological conditions.

Covalent ApDCs allow for precise delivery of therapeutics, preventing premature release of the drug and enhancing drug efficacy. Covalent linkers are used to generate covalent bonds between aptamers and drugs, such as disulfide linkers, hydrazone linkers and peptide linkers. Experimentally, conjugating drugs to aptamers via linkers can pose several difficulties. Disulfide linkers are sensitive to the intracellular reducing environment, which can lead to premature drug release before reaching the target site [98]. Hydrazone linkers are pH‐sensitive and need to maintain stability at physiological pH while ensuring cleavage in the acidic pHs like that of the tumor microenvironment (TME) [99, 100]. Peptide linkers in ApDCs can degrade due to enzymatic action, which varies with the specific enzyme levels in different tissues or tumors [100]. These complexities demand careful design and precise optimization in the creation of ApDCs to ensure they function effectively within the diverse biological conditions they encounter in the body. Cleavable linkers are designed to be stable extracellularly, but degrade under specific internal conditions, such as low pH, high glutathione or the presence of certain enzymes [101].

There are 2 studies that involve ApDCs used for GB therapy, Vorobyeva et al. [80] and Zhao et al. [88] Vorobyeva et al. developed an aptamer‐functionalized boron cluster using a boron neutron capture therapy (BNCT) agent [80]. BNCT is a radiation therapy that irradiates stable boron‐10 isotopes by neutrons and selectively destroys only ^10^B‐containing cells. GL44 is the aptamer used, which specifically targets U‐87 MG cells. They used Closo‐Dodecaborate, a non‐cleavable linker, which was attached to the 5'‐end of the aptamer via covalent bonding (Azide/Alkyne chemistry, Table 2) (B_12_‐GL44). The conjugation technique used in this research allowed for internalization of the boron‐loaded aptamer into the cells, showing potential for more efficient and less invasive treatment as a BNCT agent [80]. The only clinically used boron compounds for BNCT are boronophenylalanine (BPA) and sodium borocaptate (BSH), however, they lack effective tumor uptake on their own [102]. The xCELLigence real‐time cell analysis (RTCA) was used to assess the viability of U87‐MG cells in terms of cell index (CI; a measure of cell viability) with boron‐containing conjugates at a concentration of 1.7 µM under irradiation from a BINP neutron source. After 175 h, U87‐MG cells pretreated with B_12_‐GL44 and radiation had the lowest CI, whereas the non‐irradiated cells treated with B_12_‐GL44 had the same CI as the untreated control non‐irradiated cells. It is clear from this outcome that aptamer targeting, and neutron irradiation must be used in combination to achieve the highest treatment efficacy. These results provided insight into the tumor targeting capability of the B_12_‐GL44 system as demonstrated by its ability to distinguish between tumor and non‐tumor cells by only binding to tumor cells. This shows why adding an aptamer to a drug has great potential for the treatment of GB. The next step for this research would be to test it on a mouse tumor model.

**TABLE 2:** Table displaying the conjugation methods used in the aptamer delivery systems for GB. This table shows the types of bonds formed, their strengths and weaknesses of the conjugation chemistry and their use in vivo.

Conjugation chemistry	Type of bond formed	Strengths	Weaknesses
Aldehyde/Amine Chemistry	Imine bond	Chemically stable: highly resistant to heat, pH changes and hydrolysis. Structural rigidity Standard chemical synthesis	Enzymatically fragile. Poor Membrane Permeability Rapid Clearance
Streptavidin/biotin interaction	Non‐covalent interactions (hydrogen bonding and Van der Waals interaction)	Low non‐specific binding High affinity—one of the strongest non covalent bonds Tetrameric structure	Immunogenicity from the streptavidin. Can cause high accumulation in organs over the tumor target The bond is so strong it is hard to cleave to release the drug.
Redox‐cleavable Disulfide Bonds	Disulfide Bonds	High physiological selectivity S‐S bonds utilize the body's natural redox potential to trigger drug release.	Cross reactivity by disulfide exchange. Premature cleavage in blood circulation.
Amphiphilic Molecule Bridge	A compound with both hydrophilic and hydrophobic regions interacting	Can encapsulate both hydrophilic and lipophilic drugs. From supramolecular architectures that protect against degradation.	Upon dilution, these structures readily fall apart. Premature drug release Sensitivity to salt and pH
RNA Annealing	ssRNA molecules pair up via hydrogen bonds to form a double‐stranded helix	High specificity Generally non toxic	Susceptible to degradation Interference by RNA secondary structures.
Azide/Alkyne Chemistry	1,2,3‐Triazole	Simple synthesis Fast kinetics Mild conditions Good pharmacokinetics in vivo	If synthesized using copper catalysts—trace copper residue can be toxic. Off target interactions.
DNA Annealing	ssDNA molecules pair up via hydrogen bonds to form a double‐stranded helix	High specificity Generally non toxic	Susceptible to degradation Interference by secondary structures.
Thiol/Malemide Chemistry	Covalent thioether linkage	Fast reaction kinetics Mild conditions Commercially available	Bond is not highly stable in vivo Premature drug release
Carboxylic acid/Amine Chemistry (EDC/NHS)	Amide bond	Highly stable covalent linkage Aqueous compatibility Simple synthesis	Unreacted EDC is cytotoxic and can denature proteins Self‐conjugation with proteins (‐COOH and ‐NH2 groups). Strict pH dependency for reaction.

The second study, Zhao et al. [88] created an aptamer drug conjugate by linking HG1‐9 and highly potent anti‐tubulin drug monomethyl auristatin E (MMAE). A cathepsin‐sensitive cleavable linker valine‐ citrulline (VC) dipeptides was chosen which is stable in serum but susceptible to cleavage by cathepsin B within the lysosome of tumor cells (Thiol/Maleimide chemistry, Table 2). This ensures the ApDCs releases the MMAE and can achieve anti‐tumor effect. A transwell model was used to check the permeability of the ApDCs through bEnd.3 cells to reach U‐87 MG cells, showing penetrating ability through a BBB model. Using an orthoptic U‐87 MG mouse model after intravenous (IV) administration every 4 days there is evidence of complete suppression of tumor growth with survival going from 48 days to 61 days with treatment. Both studies show great potential for the ApDCs to be used for GB, some different combinations could be explored. The potential downside of ApDCs is that the drug is not protected it is vulnerable to nuclease degradation leading to a shortened half‐life and rapid renal clearance due to their small size [103].

#### 3.1.2 Aptamer‐Nucleic Acid Conjugate

Nucleic acids can act as therapeutic drugs such as messenger RNAs (mRNA), small interfering RNAs siRNA, microRNAs (miRNA) and therapeutic aptamers. All of which apart from aptamers need a drug delivery system to deliver them in vivo [104]. Most of these drugs regulate gene expression, offering a broad range of targets. This works well for genes with defective protein that are different to target with conventional small molecule drugs [105].

Affinito et al. [83] developed a chimera to deliver miRNA (miRNA‐34c and anti‐miRNA‐10b) using A40s aptamer (patient derived GSCs). A molecular duplex chimera of microRNA‐34c cargo (A40s‐miR‐34c) and a chimera conjugate with anti‐miRNA‐10b (A40s‐anti‐miR10b) were created using sticky‐end annealing with A40s (RNA annealing, Table 2). The A40s chimera was delivered to the stem population and not the differentiated cells, showing specific targeting despite this, further in vitro and in vivo studies must be conducted; specifically, the impact of the BBB, as well as the biodistribution and anti‐GB effect of the A40s aptamer as a delivery vehicle in the system. This study shows the aptamer being the targeting ligand and the delivery system in one, which is a smart way to use it dually and keeps the drug delivery system small. The potential downfall is that the drug no longer has protection from degradation, which may decrease the dosage reaching the brain.

### 3.2 Aptamers as Targeting Agents

#### 3.2.1 DNA Nanostructures

Nucleic acids can be synthesized to be a drug delivery system themselves. They can be created into nanostructures such as origami, tetrahedron and nanotubes, normally self‐assembling. Nucleic acids offer high biocompatibility, precise size control and minimal off‐target toxicity [106]. Cells can uptake structured DNA if the size and morphology is compatible [107]. DNA tetrahedra and triangular prisms can interact with scavenger receptors which helps with uptake. Cellular uptake can be increased by conjugating small molecules or macromolecules (aptamer or peptides) to the surface of the structure as well as spatially organizing the ligands [108, 109]. DNA nanostructures work well as delivery vehicles in the brain because they are small enough to pass through physiological barriers like the BBB and their compact rigid structures allows for effective tissue penetration. They also are biocompatible, biodegradable with minimal cytotoxicity, maybe they are great drug carriers for GB therapy.

Fu et al. [75] synthesized a tFNA loaded with temozolomide and conjugated with AS1411 (tumor penetration) and GS24 (BBB penetration) aptamers (DNA annealing, Table 2). The lethality of the system was tested on 4 different cell lines A172, U‐87 MG, T98G and LN‐18. The system killed A172 and U‐87 MG better than using temozolomide alone. The system managed to reduce drug resistance in temozolomide‐resistant cells (T98G and LN‐18) via downregulating the expression of O6‐methylguanine‐DNA‐methyltransferase. In vivo studies on healthy BALB/c nude mice showed that functionalized tFNA could penetrate the BBB, infiltrate the brain parenchyma, and remain in the brain for at least 1 h following tail vein injection. Further in vitro and in vivo studies must be conducted on the tFNA‐TMZ system, including in vitro BBB and tumor spheroid studies, to evaluate its penetration abilities, in vivo tumor inhibition and median survival rate, to fully understand the anti‐tumor efficacy of this system.

Wang et al. [92] developed a DNA tetrahedron loaded with doxorubicin and conjugated (DNA annealing, Table 2) with Gint4.T (DOX@Apt‐TDN). DNA is negatively charged, thus making it difficult for it to cross the negatively charged cell membrane, however, after 3 h incubation with Apt‐TDN, there was a significant increase in TDN uptake by U‐87 MG cells compared to unmodified TDN. Similarly, the cellular uptake of DOX@Apt‐TDN was superior to that of DOX@TDN and had a twofold increase in intracellular DOX to that of the free DOX treated cells. The apoptosis rate of DOX@Apt‐TDN was also highest, suggesting the aptamer had specificity for U87‐MG cells. These results indicate enhanced targeting and drug delivery of Gint4.T modified nanocarriers but require further in vivo testing to confirm and understand its capacity to penetrate the BBB.

Shi et al. [82] designed a tFNA loaded with paclitaxel and conjugated (DNA annealing, Table 2) with aptamers GMT8 and Gint4.T. They are formed from self‐assembled tFNA of 4 strands including GMT8 and Gint4.T. In vitro studies showed that aptamer tFNA was internalized approximately 40% more into U‐87 MG cells and had a much higher BBB penetration ability than tFNA when using the BBB transwell model (U‐87 MG/bEnd.3 cells). The aptamer tFNA had higher cytotoxicity, promoting apoptosis and significantly stronger inhibition on cell migration and invasion in U‐87 MG cells when compared to tFNA and paclitaxel alone. Despite the preliminary results indicating that this system has an anti‐GB effect, further in vivo studies must be conducted to corroborate these in vitro results.

Seo et al. [78] developed a DNA‐protein hybrid nanosphere constructed of the self‐assembled ssDNA containing biotin molecules which encapsulate doxorubicin. Streptavidin was added, conjugating to the biotin and allowed for the conjugation of the AS1411‐biotin aptamer. This nanosphere is efficiently synthesized, structurally stable, had high drug loading, has biocompatibility and is biodegradable. They proved that the aptamer targeted nanospheres were selective for GB cells (U‐87 MG and U‐251 MG) and there was improved drug penetration. The system was checked on a transwell model with hCMECs/U‐87 MG cells, the free doxorubicin was quicker to pass through the BBB model than the AS1411‐nanosphere at 30 min, however after 1 h the concentration of free doxorubicin is much less and is higher from the Dox‐AS1411‐nanosphere up to 5 h, showing a better control release profile. The anti‐tumor effect was tested in U‐87 MG tumor bearing mice, the Dox‐AS1411‐nanosphere showed to decrease the volume of the tumor, however it was not statistically significant. This delivery system has potential as shown by the data but will need more development to make a significant anti‐tumor effect.

These studies lack in vivo data; this would help decide if this type of drug delivery system should be explored further. As the biodistribution of nucleic acids can be different from other delivery systems because of the negative charge.

#### 3.2.2 Nanoparticle

Nanoparticles (NPs) exhibit excellent biocompatibility with tissue and cells across various types such as inorganic, organic, lipid‐based, protein‐based, and nucleic acid‐based NPs [110]. Aptamer‐conjugated NPs (Apt‐NPs) have demonstrated significant progress across multiple medical fields, including oncology, inflammatory diseases and viral infections [111].

Apt‐NPs act as a dynamic platform for targeted delivery and diagnostics, capitalizing on their ability to selectively bind to specific targets. This specificity reduces the risk of off‐target effects and enhances the delivery of therapeutic agents directly to cells, improving treatment efficacy while minimizing harm to healthy tissues. Additionally, the controlled release properties of Apt‐NPs ensure a steady, localized release of drugs, which optimizes therapeutic outcomes and reduces systemic toxicity. Their small size and low immunogenicity further facilitate cell penetration and decreases immune system activation [112]. This multifunctional approach holds significant promise for advancing the precision and effectiveness of brain therapies. Ten research papers explored different types of Apt‐NPs for treatment of GB, including Metal‐NP, Silica‐NP and Polymeric‐NP.

##### 3.2.2.1 Metal NP

Three different types of metal‐NPs were used, Mesoporous Ruthenium NP, Au NP and Fe_3_O_4_ NP. Metal NPs are considered excellent advanced delivery systems due to their unique physicochemical properties, which cannot always be achieved with organic carriers. They can defeat biological barriers and allow for controlled release of therapeutics with minimal toxicity [113].

Zhu et al. [76] bound transferrin and AS1411 aptamer via redox‐cleavable disulfide bonds (Table 2) on the surface of mesoporous ruthenium NPs (MRN) loaded with anti‐tumor drug [Ru(bpy)_2_(tip)]^2+^ (RBT) to create RBT@MRN‐SS‐Tf/Apt. Designed to combine glutathione‐responsive drug release with photodynamic therapy (PDT) to enhance GB treatment, this nano‐system is a relatively unique photo‐activatable formulation. The release of RBT is stimuli dependent on GSH levels, as they are much higher in tumor cells than in normal cells, and function as a photosensitizer in PDT. In PDT, the anti‐tumor drug would generate reactive oxygen species (ROS) when under a specific wavelength irradiation and consequently induce targeted tumor cell apoptosis. Even with the increased size RBT@MRN‐SS‐Tf/Apt after the addition of the aptamer it still had a significantly higher cellular uptake efficiency in U‐87 MG cells. They next tested the model on a U‐87 MG spheroid model which utilized HBMEC monolayers to model the BBB, and tumor spheroid models to model the TME, demonstrating that dual‐functionalized RBT@MRN‐SS‐Tf/Apt was superior in migrating across the BBB and penetrated deeper into tumor spheroids than the other control models. In U‐87 MG tumor bearing mice RBT@MRN‐SS‐Tf/Apt had significantly higher intensity in the brain than the non‐aptamer groups after 24 h and the RBT@MRN‐SS‐Tf/Apt along with PDT had significant GB inhibition effects. RBT@MRN‐SS‐Tf/Apt combined with PDT also allowed for an increased median survival time from 40 days to 56 days. For this delivery system, the PDT seems to be the main reason for the successful outcome over using the targeted aptamers.

Sathiyaseelan et al. developed a AS1411 (Apt) functionalized bio‐synthesized gold NP (AuNP) based delivery system with 5‐fluorouracil (5FU) and Dox loaded chitosan (CS) NPs (Apt‐Dox‐CS‐Au‐5FU NPs) for GB treatment [77]. The AS1411 aptamer functionalized Dox‐CS‐Au‐5FU NPs were conjugated using Carboxylic acid/Amine chemistry (EDC/NHS) (Table 2). In vitro studies showed that Apt‐Dox‐CS‐Au‐5FU NPs had higher cellular uptake, cytotoxicity, and lower necrosis of LN229 cells than the non‐aptamer NPs. The pH‐responsive nature of CS allowed Apt‐Dox‐CS‐Au‐5FU NPs to induce apoptotic cell death without causing mitochondrial loss or necrosis related inflammation because of the controlled drug release caused by the CS coated Au‐5FU NPs. Despite the enhanced in vitro response, further in vivo GB studies are required to determine passing of the BBB, the specific targeting effect and the anti‐cancer effects of this nanocarrier system. The aptamers used have enhanced the metal‐NP anti‐tumor effects in all studies, showing using aptamers in drug delivery systems for GB is favorable.

##### 3.2.2.2 Silica NP

Mesoporous silica nanoparticles (MSNs) have transformed the field of controlled drug delivery systems. They have great features that make them well suited to this application. They have well‐ordered internal mesopores with large pore volume (high drug loading), great surface area and their size can be changed to fit what needs to be achieved. They are robust and can be easily modified. This makes them great multifunctional nano systems [114].

Fei et al. [91] created a Gint4.T‐siHDGF chimera‐capped MSN encapsulating temozolomide (TMSN@siHDGF‐Gint4) (Aldehyde/amine chemistry, Table 2), delivering temozolomide and siHDGF, as the combination of siRNA‐based gene therapy and chemotherapy has great potential. This system has spherical nucleic acid‐like architecture which can enhance the enzyme resistance of siHDGF and increases the BBB crossing. The addition of Gint4.T allows for GB cell specific binding. The benzoic‐imine bond on MSN surface is cleaved in the acidic lysosome environment (pH sensitive), which leads to the rapid release of the chimera which leads to the gene silencing of HDGF. This is then followed by the slower release of temozolomide from the MSN to increase cytotoxicity. They demonstrated the crossing of the BBB using a transwell model with bEnd.3 cells as the BBB and U‐87 MG cells as the GB model. In vivo, they used U‐87 MG tumor‐bearing mice, after administration of TMSN@siHDGF‐Gint4.T the tumor growth is slowed down and the survival time of the mice increased from 24.5 days (untreated) to 58 days. This data shows great promise for this platform for a targeted dual treatment approach using a silica‐NP for GB.

##### 3.2.2.3 Polymeric NP

Biodegradable and biocompatible polymers are mainly used to produce polymeric‐NP such as poly(lactide) (PLA), poly(ε‐caprolactone) (ε‐PCL) and poly(lactide*‐co‐*glycolide) (PLGA) [115]. Biopolymers are also used such as chitosan or collagen use to their non‐immunogenic properties, which are hydrolyzed into harmless products once the drug is released. Polymeric‐NP are advantageous for drug delivery because they can be modified to suit the needs of the system such as the size is tunable, they are normally small in size (∼70 nm) helping them pass the BBB, they protect the cargo they are carrying, they control drug release, they are highly biocompatible and safe in the body [116]. When adding a targeting aptamer to the surface, it will make them even more selective, allowing for targeting of the BBB and the tumor. Positively charged NPs readily bind to the negatively charged endothelial cells of the BBB, however, they can cause cytotoxicity and rapid immune clearance. Negatively charged NPs are potentially a safer alternative; they minimize non‐specific protein adsorption, less toxicity and they enhance the formation of protein coronas that naturally utilize endogenous BBB transport mechanisms. As aptamers are negativity charged the polymeric‐NP will move to have a slightly negatively charged surface once conjugated. The addition of BBB targeting aptamer will also allow for receptor‐mediated transcytosis [117].

Gao et al. [81] used GMT8 to design a functionalized PEG‐PCL NP (ApNP) loaded with docetaxel. GMT8 was conjugated to the surface via carboxylic acid/amine chemistry (EDC/NHS) (Table 2). Cellular uptake studies in U‐87 MG cells demonstrated that ApNP significantly enhanced uptake and was GMT8 receptor mediated, as at a high dose the cells become saturated. GMT8 enhanced the transport of the particles to the core of U‐87 MG tumor spheroid models and when loaded with docetaxel it has the maximum inhibitory effect on the tumor growth compared with control groups. In U‐87 MG tumor‐bearing mice there is a 1.81‐fold increase in ApNP accumulation in the tumor site compared to untargeted NPs. The survival time was also increased from the untreated to nearly double (22 days to 40 days) for the ApNP, and the untargeted NPs have a survival rate of 35 days. The increased survival time will be due to higher accumulation of the chemotherapeutic drug in the tumor as there will be less off targeted delivery. This model has great promise for the treatment of GB. A chemotherapeutic drug along with a targeted delivery system is a good method.

Monaco et al. [93] developed a polymeric‐NP with poly(lactic*‐co‐*glycolic)‐block‐polyethylene glycol (PLGA‐b‐PEG) copolymer which encapsulated P13K‐mTOR inhibitor, dactolisib. This was conjugated with Gint4.T using Carboxylic acid/Amine chemistry (EDC/NHS) (Table 2) to create a targeted polymeric‐NP, 1@PNOs‐Gint4.T. 1@PNOs‐Gint4.T were internalized in U‐87 MG cells (which express PDGFR ß) after 10 min, while incubation with the scrambled aptamer polymeric‐NP (1@PNPs‐SCR) were still undetected after 50 min, showing great specific targeting. The cytotoxicity of dactolisib was fourfold more effect in 1@PNOs‐Gint4.T compared with 1@PNPs‐SCR, demonstrating that the Gint4.T modification improved bioavailability of the drug. In U‐87 MG tumor‐bearing mice, after 2 h post IV injection, 1@PNOs‐Gint4.T can be observed in the tumor, whereas the negative control could not be. Further in vivo studies to assess the drug delivery effectiveness of Gint4.T showed that phospho‐4EBP1 levels were significantly lower in 1@PNOs‐Gint4.T treated tumors than control tumors after systemic administration over 5 days. These results prove that the Gint4.T system can cross the BBB, accumulate in the tumor, and specifically deliver the chemotherapeutic drug by recognition of PDGFR ß expressed cells. To establish if this could potentially be a suitable treatment that would increase survival, further in vivo studies are warranted to determine how the 1@PNOs‐Gint4.T system could impact survival rate in GB tumor‐bearing mice.

Smiley et al. [64] developed a tunable polymer‐micellar NPs co‐loaded with temozolomide and RG7388 and functionalized with both a CD133‐A15 aptamer and Zirconium‐89 (^89^Zr) (Carboxylic acid/Amine chemistry (EDC/NHS, Table 2). RG7388 from the nutlin class has been proven to bind specifically to mouse double minute 2 (MDM2), which binds to P53 tumor suppressor protein leading to removal of P53 and reduction of tumor suppression. The inhibition of MDM2 would stabilize P53 presence, reduce oncogenesis and potentially offer therapeutic benefit. Temozolomide efficacy can be enhanced by addition of an inhibitor of the GB DNA damage response (DDR) system, which would help overcome GB resistance. Using temozolomide together with RG7388 seems advantageous. ^89^Zr, a gamma emitting radiotracer that allows for a theragnostic option for in vivo localization studies using PET imaging, was covalently bonded to the NP. The CD133‐A15 aptamer was bound to the NP through click chemistry of NHS‐amine reaction. In vitro studies found that CD133‐A15 conjugated temozolomide+RG7388 NPs had a higher CSCs killing effect than empty NPs and temozolomide ‐loaded NPs. The CD133‐A15 conjugated TMZ+RG7388 also showed a trend toward higher GB CSC cytotoxicity than non‐CD133 conjugated TMZ+RG7388 NPs. Despite the ability of the CD133 aptamer system to target and kill CSCs, additional in vitro and in vivo localization studies are required to determine the efficiency of CSC targeting. To fully validate the system, extensive in vivo studies must also be conducted to assess BBB penetration.

Behrooz et al. [67] develops PAMAM G4C12 dendrimer nanoparticles, formulated for the co‐delivery of paclitaxel and temozolomide. The CD133‐B19 aptamer was conjugated to the G4C12 dendrimer through maleimide click chemistry, using a thiol modifier on the aptamer, resulting in 230 nm drug‐loaded Aptamer‐NPs able to undergo nonphagocytic internalization due to its small size (<300 nm). In vitro studies revealed that Aptamer‐NPs had a high accumulation in U‐87 MG cells, and the drug‐loaded Aptamer‐NPs had a dramatically elevated apoptosis from 37.4% to 73.3% when compared to simultaneous administration of paclitaxel and temozolomide due to its CD133 targeting abilities of the CD133‐B19 aptamer. The significant enhancement of apoptosis by the drug‐loaded Aptamer‐NPs may be due to the downregulation of autophagy‐related gene expression, MDR pathway genes, biomarkers (CD133, CD44, and SOX2) and Wnt/ß‐catenin pathway when compared to PTX‐TMZ with no carrier. Although these preliminary results indicate that this nanocarrier co‐delivery system could selectively target U‐87 MG cells and potentially overcome the drug resistance in U‐87 MG stem cells, further in vivo studies are required to confirm these initial findings and show the true potential of this system against the BBB and biodistribution around the body.

Luo et al. [74] developed an AS1411 functionalized poly(l‐γ‐glutamylglutamine)‐paclitaxel nanoconjugate (AS1411‐PGG‐PTX). The AS1411 aptamer was functionalized to the surface of the nanoconjugate system using EDC/NHS chemistry (Table 2) with a conjugation efficiency of 89.8%. In vitro cellular uptake studies on U‐87 MG cells showed that AS1411‐PGG‐PTX had a threefold increase in accumulation compared to PGG‐PTX nanoconjugates at the same concentration, similar results were also seen in neo‐vascular endothelial human umbilical vein endothelial cells (HUVECs). These results indicate that AS1411‐PGG‐PTX significantly increased cellular uptake by targeting both GB and neo‐vascular endothelial cells. Further in vitro studies on U‐87 MG tumor spheroids revealed that the AS1411‐PGG‐PTX had a 2.4‐fold increase in penetration compared to unmodified PGG‐PTX due to the AS1411‐nucleolin mediated endocytosis and smaller particle size, ultimately resulting in superior diffusion into tumor spheroids. In addition to superior internalization, AS1411‐PGG‐PTX significantly increased cell apoptosis and cytotoxicity of U‐87 MG cells compared to PGG‐PTX. These findings were corroborated in vivo, whereby ex vivo fluorescence images of intracranial U‐87 MG‐PMT48‐luc xenografts in mice showed the fluorescent intensity of AS1411‐PGG‐PTX was 2.2‐fold that of the PGG‐PTX at the tumor site after 24 h. GB slices of AS1411‐PGG‐PTX treated tumors showed the modified nanoconjugate penetrated deeper into the entire GB tissue than the PGG‐PTX treated tumors, which further supports the initial in vitro tumor spheroid studies. The AS1411‐PGG‐PTX nanoconjugates had enhanced anti‐GB efficacy as the median survival time of intracranial U87‐MG GB‐bearing nude mice treated with AS1411‐PGG‐PTX nanoconjugates was 52 days versus the 47 days seen in PGG‐PTX nonconjugate mouse group. This prolonged survival time will be due to the targeted delivery of PTX to GB tumors and the extended blood circulation time caused by the smaller size and relatively neutral zeta potential because of the AS1411 modification. Overall, the addition of the AS1411 aptamer showed superior anti‐GB efficacy, cellular uptake, and tumor penetration than the unmodified PGG‐PTX nanoconjugates, which indicates the potential of aptamers to improve GB treatments.

Aptamer polymeric‐NPs have shown great promise as drug delivery systems for GB. All 5 studies demonstrated that polymeric‐NP are very diverse and can be modified to suit their delivery needs. Adding the aptamer to the surface of the nanoparticle increases the success of the delivery system in all studies.

#### 3.2.3 EVs

Exosomes, micro‐vesicles and apoptotic bodies have been reported as three major subclasses of extracellular vesicles (EVs). Exosomes, ranging from 30 to 150 nm in diameter, have attracted widespread attention in translational and precision medicine [118]. These smaller EVs are generated intracellularly within endosomal structures of multivesicular bodies (MVBs), which are vesicles that participate in the endocytic pathway. Exosomes transfer various cell constituents such as protein, lipids, DNA, and RNA between cells, playing a crucial role in intercellular communication. This process can influence various disorders at physiological and pathological levels, including cardiovascular diseases, cancer progression and neurodegenerative diseases [119]. There are ∼376 clinical trials investigating exosome applications, with the majority of studies using exosomes as biomarkers, drug delivery systems or cancer treatments.

Several research groups are actively working on functionalizing exosomes with aptamers to further enhance drug delivery efficacy. In these processes, the encapsulation of therapeutics can be completed via incubation methods, electroporation methods, transfection of parent cells, and surface functionalization [120]. Aptamers covalently or non‐covalently conjugated to exosomes are being investigated. Non‐covalent methods, such as electrostatic interactions and lipid insertion, maintain the biological activity and structure of exosomes, making them ideal for diagnostic applications where exosome integrity is essential. Zou et al. have developed a non‐covalent method for conjugation of aptamers with exosomes [121]. Specifically, aptamers modified with a hydrophobic diacyl lipid tail are anchored onto exosome surfaces via hydrophobic interactions between the diacyl lipid tails and the phospholipid bilayer of exosomes. This method leverages the natural affinity between lipid structures to attach the aptamers to the exosomes, preserving the integrity and functional properties of both components. This study demonstrated that aptamer‐functionalized exosomes specifically bind to target cells at low temperatures (4°C) and are actively internalized into these cells at normal body temperature (37°C) through a clathrin‐mediated endocytosis pathway. Through altering the usual pathways through which exosomes are taken up by cells, aptamers can enhance targeted cellular interactions. Covalent techniques, such as chemical crosslinking using EDC/NHS, ensure robust and stable attachment of aptamers to exosomes, crucial for enduring the diverse physiological environments in vivo.

Liang et al. [66] reports dual‐receptor‐specific exosomes as vehicles loaded with temozolomide and O6‐benzylguanine for eradicating temozolomide resistance in GB. The target ligands angiopep‐2 and CD133 RNA aptamers were conjugated on exosomes via an amphiphilic molecule bridge (Table 2), which was induced to express on donor cells. The dual targeting allowed BBB penetration and then selective uptake of the vehicle into the desired tumor cells. IV administration of the vehicles in U‐87 MG tumor‐bearing mice resulted in twofold increase in accumulation compared to the non‐targeting exosome. Therapeutically, in vivo the targeted exosome increased survival time of the animals by 43.75% compared to the free drug. These targeted exosomes provide a promising system for GB therapy.

An exosome is a natural biological phospholipid nanovesicle characterized by low toxicity, high biocompatibility, low immunogenicity, high blood stability as well as BBB penetration capabilities. Due to their ability to penetrate the BBB they provide a perfect platform to then be modified with a tumor targeting aptamer, more Aptamer‐EV GB delivery systems should be studied.

#### 3.2.4 Liposomes

Liposomes are spherical vesicles that consist of one or more phospholipid bilayers, which enable them to encapsulate a variety of therapeutic agents. Due to their stability, biocompatibility and biodegradability, liposomes have been studied and utilized as a powerful drug delivery system [122]. In clinical settings, liposomes are primarily employed in the treatment and diagnosis of cancer. Although liposomal formulations are effective as drug carriers, due to the presence of various cancer and immune cells, tissue selectivity at tumor sites is difficult to achieve when solely reliant on passive permeation and retention [123]. To overcome this limitation, several research groups have explored conjugation of aptamers to liposomes in order to improve selective drug efficacy within the tumor microenvironment. Integration of aptamers with liposomes can be achieved through both non‐covalent and covalent methods of coupling. Although cationic liposomes can facilitate non‐covalent binding because of their positive charge, covalent attachment is generally preferred when forming stable aptamer‐liposome conjugates due to their stronger affinity and lower susceptibility to environmental changes such as pH, temperature or ionic strength. Direction strategy, pre‐conjugation strategy and post‐insertion strategy are three common ways of generating aptamer‐liposomes conjugates [124]. The most commonly used and successful method for conjugating aptamers to liposomes is the post‐insertion strategy. This method involves first chemically linking aptamers to lipids to create aptamer‐lipid conjugates, which are then incorporated into pre‐formed liposome bilayers through incubation [125].

Sun et al. [65] uses a dual modified cationic liposome loaded with survivin siRNA (inhibitor of apoptosis, highly expressed in GB tissue) and paclitaxel. It was dual conjugated with angiopep‐2 and CD133‐A15 aptamer for BBB penetration followed by tumor cell targeting. The targeting ligands were conjugated to the liposome via the COOH group on the PEG to the DSPE group on the ligands using EDC as a coupling reagent (Carboxylic acid/Amine chemistry (EDC/NHS), Table 2). This delivery system was tested in U‐251 MG‐CD133+ tumor bearing mice. The targeting liposome had superior uptake compared to the untargeted liposome, facilitating the delivery of survivin siRNA as seen in the significant decrease of survivin mRNA levels in U25‐CD133^+^ cells. The medium survival rate of tumor bearing mice after therapy was 81 days versus 41 days for the non‐targeted liposome therapy.

Adding BBB and GB targeting ligands onto the liposomes is a productive way to help it cross the BBB and target the tumor cells specifically. More liposome aptamer conjugates should be tested for GB therapy as this study shows promise along with other studies that target other types of cancers.

#### 3.2.5 Emulsion

Microemulsions are clear, thermodynamically stable, isotropic liquid mixtures (oil, water and surfactant). Their size is typically 5–100 nm, they form spontaneously and offer high solubilization of hydrophilic and lipophilic compounds [126]. Microemulsions can increase the solubility and bioavailability of poorly soluble drugs, improve permeation and provide sustained and targeted drug release across different administration routes. This is why they are great potential drug delivery systems. Oil phase components can increase the affinity of drugs to BBB endothelial cells and surfactants/cosurfactants can inhibit or reduce P‐glycoprotein efflux [127, 128]. If then conjugating targeting ligands to these systems, it will help deliver the payload with more accuracy. The common ways to conjugate these are via ligand‐coupled surfactant techniques and covalent bonding (NHS‐ester or maleimide‐thiol).

Wang et al. [72] synthesized a hyaluronic microemulsion which encapsulated shikonin and docetaxel. Both drugs have limited absorption and anti‐glioma efficacy, due to their inability to cross the BBB. The emulsion is conjugated aptamer AS1411 to its surface via thiol/maleimide conjugation (Table 2). The increase of uptake of the emulsion in U‐87 MG cells after the addition of AS1411 was up 38.8%. The addition of AS1411 added no significant difference to the penetration of the BBB when using U‐87 MG/CSC spheroids model, the small size of the microemulsion is already allowing it to cross. However, therapeutically the tumor was 16.9‐fold decrease in tumor spheres compared with non‐targeted emulsion (4.5‐fold decrease). During the survival study, the non‐targeted microemulsion the mice survived between 42–54 days. The aptamer targeted emulsion 25% of the mice survived within 60 days after treatment, confirming the needed for the AS1411 aptamer as a targeting ligand.

Wang et al. [73] developed a dual‐modified superparamagnetic Fe_3_O_4_ NP‐embedded microemulsion loaded with Shikonin and Docetaxel using AS1411 aptamer and T7 peptide (Fe_3_O_4_@T7/AS1411/SKN&DTX‐M). The T7 peptide and AS1411 aptamer were conjugated onto the microemulsion using thiol/maleimide chemistry (Table 2) which caused an increase in particle size than the unmodified microemulsion because the T7 peptide and AS1411 aptamer formed a hydration layer coating outside of the microemulsion. Although the encapsulation of Fe_3_O_4_ did not significantly affect the drug release profile, in vitro cellular uptake studies, it was revealed that dual targeted modifications (T7/AS1411/SKN&DTX‐M) increased intracellular docetaxel and apoptosis in G422 cells compared to singularly ligand or non‐ligand groups. In the G422 tumor‐bearing mouse model, the tumor size decreased for Fe_3_O_4_@T7/AS1411/SKN&DTX‐M with 25% of the mice surviving up to 90 days post implantation compared to 54 days of the control animal groups, indicative of superior tumor targeting and penetration. Aptamer‐emulsion drug delivery systems have promise to be used therapeutically for GB. Different combinations of aptamers and targeting ligands and drugs delivered could be explored.

## 4 Discussion and Future Perspectives

GB remains a universally fatal malignancy with poor prognosis. There remains a dire need for novel drug delivery systems with potent antitumoral properties. In recent decades, the range of therapies for other solid cancers, and in turn their prognoses, has drastically improved, primarily attributed to advancements in mABs. However, this trend has not been observed for GB. Confounded by size, hydrophilicity and charge, mABs (along with other standard chemotherapeutic agents) are impeded in their ability to effectively cross the BBB [129].

All studies described show using aptamers in drug delivery systems for GB therapy has great potential. The main themes are using an aptamer as the delivery system or using the aptamer as the targeting ligand in a delivery system. When using the aptamers as a targeting ligand, they are used for targeting the tumor or targeting the BBB. Some of the studies are dual targeting first the BBB and then followed by the tumor, such as using Gint4.T for PDGFR‐ β then GMT8 for U‐87 MG cells or GS24 for transferrin then AS1411 for nucleolin on the tumor. Some use 1 aptamer and 1 other type of targeting ligand such as T7 peptide for transferrin followed by AS1411 for nucleolin and angiopep‐2 peptide for LRP1 and CD133‐A15 for the CD133 surface protein. Eleven studies use tumor targeting aptamers, 4 studies use BBB targeting aptamers, 2 studies use 2 aptamers for BBB and tumors, and 2 studies use 1 peptide for BBB targeting along with 1 aptamer for tumor targeting.

The chosen targets on the tumor have been overexpressed cell surface markers (CD133, nucleolin) or whole tumor cells (U‐87 MG or patientderived GSCs). Other surface markers that could be explored are EGFR, p53 and EphA2 receptors. EGFR is overexpressed in ∼60% of GB tumors and is the primary driver of tumor cell proliferation. It has not been that successful as a treatment alone, but the overexpression may make for a potential target [130]. Coupling an EGFR aptamer to a drug‐loaded NP system would be an elegant therapeutic strategy. Thomas et al. [131] recently investigated the use of an anti‐EGFR RNA aptamer, MinE07, for the treatment of non‐small cell lung carcinoma (NSCLC)) and found that MinE07 bound with high affinity to wildtype EGFR and demonstrated superior labelling of NSCLC when used in conjunction with other aptamer formulations. Given the success of MinE07 within NSCLC, its use as a delivery system within GB has potential [131]. Driven by the presence of p53 mutations within GB, conjugating p53 aptamers to drug‐loaded NPs may prove to be another effective therapeutic approach. Kong et al. used a DNA aptamer, termed dp53m, targeting the p53‐R175H mutant protein associated with many cancers. Dp53m induced selective proteasomal degradation of p53‐R175H, whilst preserving wildtype p53 in a pharyngeal cancer cell line, Detroit 562, as well as exhibiting a synergistic relationship with cisplatin [132]. These findings lend support toward further investigations of p53 aptamers in the context of GB, which can sensitize GB to traditional chemotherapeutic agents, especially as resistance to temozolomide is commonly observed. The EphA2 receptor is a transmembrane receptor tyrosine kinase which plays a crucial role in development, tissue homeostasis and cancer progression. It is highly overexpressed in some solid tumors, especially GB [133]. EphA2 receptor is a good GB target as it is overexpressed in ∼90% of GB patients compared to low expression in healthy brain tissue [134]. There are other types of cells unexplored inside the tumor that could be utilized for targeted delivery such as tumor‐associated macrophages (TAM) and tumor infiltrating lymphocytes (TILs). TAMs form approximately 50% of tumor mass, this would be a great target when exploring immunotherapeutic approaches [135]. Studies such as these highlight the value and potential of SELEX to identify alternative aptamers as delivery systems, such as for EGFR, p53, EphA2 and macrophages for GB treatment [136].

The chosen targets on the BBB have been cell surface markers of PDGFR‐ β (present on brain capillary pericytes) and transferrin receptors (present on brain capillary endothelial cells). Some other BBB targets that could be employed are basigin, Glut1 and CD98hc, which are all highly expressed on the brain endothelial cells [137]. Or LRP1 which is significantly overexpressed in GB brain compared with healthy brain [138]. Using a dual targeting approach such as a BBB targeting ligand to first bypass the BBB alongside a GB aptamer to then specifically target the tumor could be a good way to directly treat GB with limiting side effects.

Most of these aptamers were selected using cell‐SELEX except for GS24 which was selected by protein‐SELEX and AS1411 which was selected via a non‐traditional SELEX way of screening of G‐rich nucleotides. This shows cell‐SELEX to be the most successful method for GB aptamer discovery. Cell‐SELEX allows for the aptamers to bind to whole sections of the cells over just the protein in a non‐native state, challenging the aptamer library to a more complex environment. However, potentially cell‐SELEX was the method of choice because it is relatively cheap and simple compared with other methods, requiring only standard biological lab skills. Using automated‐SELEX or in vivo SELEX may be advantageous, as these methods allow for a more real environment including the BBB. A BBB mimicking system could be constructed in a microfluidic chip, allowing for earlier removal of aptamers that do not make it across the BBB. Using a GB tumor‐bearing model for in vivo SELEX will allow for removal of all off targeting aptamers more stringently. This will cut down the failures of moving aptamers from in vitro to in vivo.

The most common drug delivery system used with aptamer as a targeting agent was the NP, with 8 unique platforms designed using different types of materials as the NPs. Carboxylic acid/amine chemistry (EDC/NHS) or thiol/maleimide chemistry are the mostly used conjugation methods as it is easy, efficient and biocompatible (Figure 3B). The studies outlined in this review consistently highlighted the superior cellular specificity and enhanced antitumoral properties of Aptamer‐NP compared to treatment approaches without aptamer conjugation. It is clear that the synthesized Aptamer‐NPs in these studies also meet the necessary physiological requirements in terms of diameter and zeta potential. Aptamer‐NPs, given their low nanometer‐range sizes (from as miniscule as 13.5 nm regarding the DOX@Apt‐TDN study) [92], can circumvent the permeability challenges posed by the BBB and as such show great promise as an attractive therapeutic alternative for GB treatment [139]. Given their superior properties and enhanced therapeutic efficacy, further in vivo studies are warranted to ascertain the extent of BBB diffusion as this is not described in every study as well as accurately determine biodistribution and survival outcomes. Aptamer‐functionalized lipid nanoparticles (LNPs) were not explored in any study for GB therapy to date. LNPs are considered the most successful delivery system because of their biodegradability, biocompatibility, superior drug encapsulation and ability to cross the BBB. They are very advanced and FDA approved; they would be a good platform to explore including a targeting aptamer for GB [140].

**FIGURE 3:**
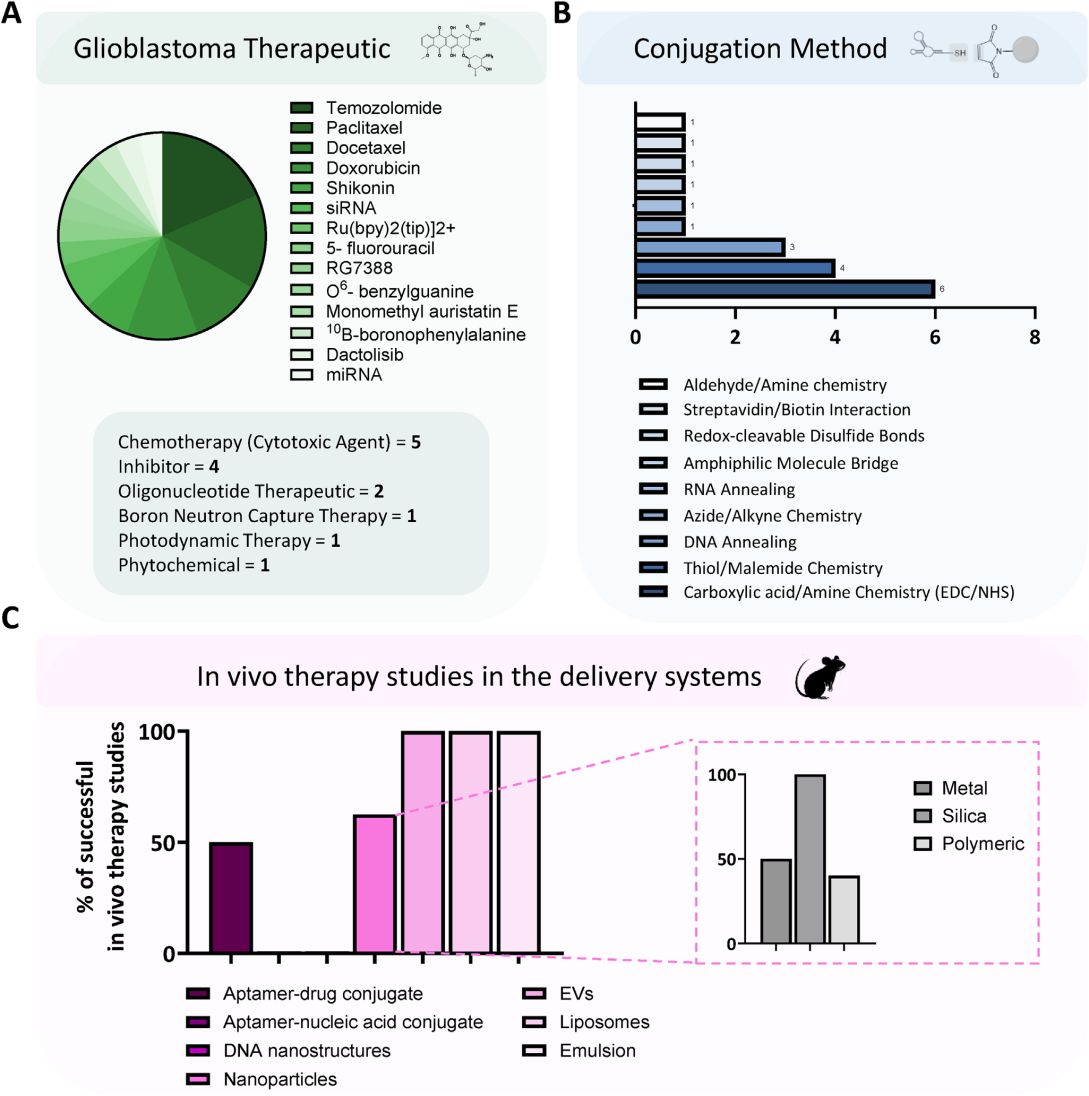
(A) A pie chart representing the therapeutic landscape in aptamer‐targeted drug delivery for glioblastoma. (B) A bar chart representing the conjugation methods used in aptamer‐targeted drug delivery for glioblastoma and their frequency. (C) Bar chart representing the % of successful in vivo therapy studies grouped by delivery system. It shows which delivery systems have transitioned from in vitro studies to in vivo successfully. The bar chart on the right‐hand side represents the break down the nanoparticle delivery system group.

The most common therapy used in these studies was chemotherapeutic drugs, temozolomide and paclitaxel. As well as utilizing aptamers to deliver chemotherapeutic agents, by conjugating aptamers to boron compounds, targeted cellular internalization can be achieved, meaning aptamers can enhance radiation therapies (such as BNCT) as well as reduce off‐target effects. AuNPs or other metallic NPs have also demonstrated potential to enhance radiotherapy, or alternatively non‐ionizing therapies such as photothermal ablation of tumor cells, for which aptamers could be utilized to enable targeted delivery. Immunotherapeutic drugs feature the least; however, this is an interesting avenue to explore. The GB tumor is known for having an immunologically ‘cold’ environment, so using checkpoint inhibitors or enhancers will help convert the tumor to a ‘hot’ environment [141]. CAR‐T therapy or the delivery of nucleic acids such as mRNA and siRNA encapsulated in an LNP could be explored for GB using a targeting aptamer to guide it to the tumor specifically (Figure 3A).

The choice of conjugation method used is very important in determining the drugs stability, the targeting precision, controlled release and the toxicity. Having the optimized conjugation type can enhance or deplete your delivery platform. Carboxylic acid/amine chemistry (EDC/NHS) and thiol/maleimide chemistry are the most commonly used conjugation methods. Both are highly effective with good yields and favorable safety profiles for moving in vivo. The amide bond formed by carboxylic acid/amine chemistry will be more robust than the thioether linkage created by thiol/maleimide chemistry. However, the advantage of the thioether linkage is that it is highly site‐specific and not cross‐reactive compared with the carboxylic acid/amine chemistry as proteins contain multiple primary amines that can randomly crosslink, potentially altering protein function. The amide bond is a permeant bond, potentially slowing down drug release, the thioether linkage breaking easier allowing for definite drug release. DNA/RNA annealing is also widely used, especially for oligonucleotide‐based systems. It is highly effective and straightforward to implement, but the resulting complexes are more susceptible to degradation, and this approach is limited to nucleic acid‐based components, making it less universally applicable than the more standard chemical bond linkages.

Translation from in vitro studies into in vivo and more humanized models is essential for the progress in pharmaceutical research. Of the studies examined 58% of them transition from in vitro cell work into GB tumor mouse models (Figure 3C). Both groups containing oligonucleotides (aptamer‐nucleic acid conjugates and DNA nanostructures) do not have any success in vivo studies. Only 1 article tested their system in vivo (DNA‐protein hybrid nanosphere by Seo et al.), showing that though Dox‐AS1411‐nanosphere showed to decrease the tumor volume, it was not statistically significant. Potentially the oligonucleotide drug delivery systems are in early stages of development with research teams not equipped to run in vivo studies or oligonucleotide drug delivery systems do not have the chemical stability offered by other types of systems. The in vivo studies produced promising data from the EVs, liposomes and emulsion groups, however the number of studies using these platforms is much smaller. To get a greater picture of these types of delivery systems a larger number of platforms would need to be tested. For aptamer‐drug conjugate and NPs approximately 50% of studies have successfully transitioned from in vitro to in vivo. BNCT is complicated in animal models, potentially delaying the process from cells to rodents. Some of the NPs studies produced good in vivo data with some studies increasing their animals survival time by 2×. NPs in vivo can have issues with biological barriers, rapid clearance and tumor penetration which the researchers have tried to overcome with the addition of a targeting aptamers, however the aptamer alone may not change the success of the therapy. The type of therapeutic used did not change whether it was successful in vivo or not, it was more due to the drug delivery carrier. The aptamers used do not correlate with the success in vivo, AS1411 aptamer being the most popular is shown to be successful in some studies and not in others, meaning the change of system is more important. Some research teams will have been unable to move their work in vivo due to a lack of support.

Most of these studies explored humanized mouse models such as U‐87 MG tumor‐bearing nude mice. The limitation of this model is their lack of T‐cell mediated immunity. For chemotherapeutic agent testing it is an adequate model, however if wanting to explore immunotherapy, an immunocompetent mouse model would be required such as GL261 or CT‐2A in C57BL/6 mice [142]. In studies that conducted in vivo investigations, Liang et al. [66] reports a dual‐receptor‐specific exosomes (CD133 aptamer and angiopep‐2 ligand) resulted in a twofold increase in GB uptake and approximately 40% increased survival times. However, given the immunogenicity posed from exosomes themselves, significant considerations must be given to how these in vivo findings will translate to the clinic, especially as the model used to explore this is immunocompromised. There are other testing models that could be explored such as organoids and tumor‐on‐a‐chip, currently creating a GB model of these has been challenging, but the creation of them will improve the GB testing platforms (Figure 4).

**FIGURE 4:**
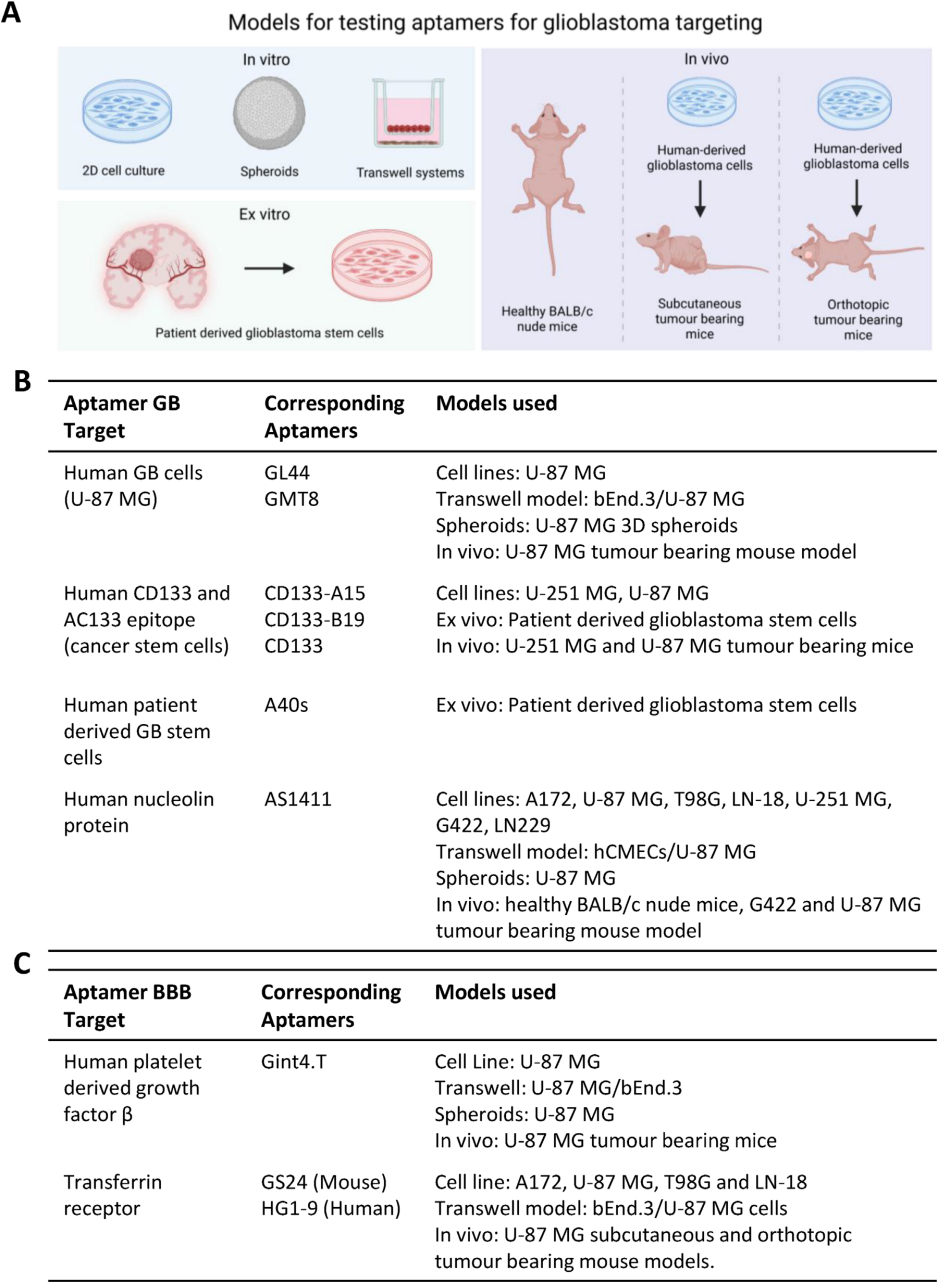
Models for testing aptamers for glioblastoma targeting. (A) Schematic of the models used to explore aptamer as targeting agents or delivery systems. (B) Table of tumor targeting aptamers and their corresponding models used. (C) Table of BBB targeting aptamers and their corresponding models used.

## 5 Conclusion

Aptamers have shown that their exceptional recognition capacity is very suitable for aiding drug delivery for GB. As described in this review, the results unequivocally show that aptamers have great potential as targeted therapeutic carriers, capable of overcoming the BBB and trafficking the tumor effectively. Researchers from around the world have successfully been able to conjugate aptamers to a diverse range of therapeutic carriers, using a wide range of conjugation strategies and chemistries. Aptamers have been shown to increase drug uptake into the correct cells and limit the loss of therapeutic payloads to non‐desirable cells. Despite the considerable benefits reported in these studies, translation from in vitro into in vivo is still needed across many of the developed aptamer‐based systems. Whilst there are key limitations and unresolved challenges, recent progress in aptamer‐based drug delivery systems has been phenomenal. In conclusion, it is clear the application of aptamers used in novel drug delivery platforms for the treatment of GB is considerable and must continue to be explored.

## Author Contributions

ARP devised the concept, conducted the research, wrote the manuscript and supervised the work. AMM, DO and YL conducted the research and wrote the manuscript. KAJ developed the concept, wrote the manuscript and supervised the work.

## Funding

ARP was supported by King's College London, University of London Maplethorpe Fellowship and Brain Research UK (202021‐30). KAJ is a Global STEM Scholar at the University of Hong Kong. Part of the work is supported by The Hong Kong Jockey Club Charities Trust. Images were drawn using BioRender software.

## Data Availability

Data sharing not applicable to this article as no datasets were generated or analysed during the current study.
